# A systematic review and meta-analysis evaluating the success of different protective liners after performing selective caries removal

**DOI:** 10.2340/biid.v12.45221

**Published:** 2025-12-29

**Authors:** Vaishnavi Ratnakar Patankar, Ashish K. Jain, Rahul D. Rao, Laresh N. Mistry, Deepakkumar Langade

**Affiliations:** a M.D.S Conservative Dentistry and Endodontics, Bharati Vidyapeeth Dental College, Navi Mumbai, Maharashtra, India; b M.D.S Pedodontics and Preventive Dentistry, Bharati Vidyapeeth Dental College, Navi Mumbai, Maharashtra, India; c Pharmacology, D Y Patil University School of Medicine, Navi Mumbai, Maharashtra, India

**Keywords:** Calcium hydroxide, dental caries, dental pulp, silicates, vital pulp therapy

## Abstract

**Aim:**

This systematic review appraised clinical trials that evaluated the efficacy of various protective liners on preserving pulp vitality after selective caries removal.

**Methods:**

This systematic review adhered to the Preferred Reporting Items for Systematic Review and Meta-analysis principles. The Population, Intervention, Comparison, Outcome and Study Design (PICOS)strategy was intended to encompass individuals with deep carious lesions in permanent teeth. Studies comparing the efficacy of calcium hydroxide with glass ionomer cement (GIC) or resin-modified glass ionomer (RMGIC) or mineral trioxide aggregate (MTA) or Biodentine or Theracal on pulp vitality after selective caries removal having 1 year of follow-up were included.

**Results:**

After full-text analysis, eight studies (*n* = 8 trials, 678 teeth) were included. The results of meta-analysis of seven studies indicated that there was no statistically significant difference between RMGIC and calcium hydroxide (risk ratio [RR] = 0.98; 95% Confidence limits (CL) = 0.92 to 1.06; *P* = 0.68), Biodentine and calcium hydroxide (RR = 1.05; 95% CL = 0.94 to 1.18; *P* = 0.40), Theracal and calcium hydroxide (RR = 1.02 ; 95% CL = 0.97 to 1.07; *P* = 0.48) and MTA and calcium hydroxide (RR = 1.13; 95% CL = 0.95 to 1.34; *P* = 0.17). Sensitivity analysis also showed no significant difference between Theracal and calcium hydroxide. Heterogeneity was moderate for comparison between MTA and calcium hydroxide (*I*² = 54%). Two studies were with low risk of bias, and six studies were classified as having some concerns. Evidence ranged from very low to moderate.

**Conclusion:**

There were no differences in clinical outcomes between RMGIC and calcium hydroxide, Biodentine and calcium hydroxide, Theracal and calcium hydroxide and MTA and calcium hydroxide. Thus, the findings suggest that treatment success is not significantly influenced by the type of protective liner employed.

## Introduction

The field of dentistry has seen substantial changes since its professional inception, with more invasive treatments being replaced by minimally invasive dentistry [1]. One such domain is the management of deep dentinal caries [2]. The early practice of complete caries removal is now slowly being replaced by selective caries removal [3]. Selective caries removal can be defined as a procedure in which carious dentin is removed completely from the periphery while leaving behind a layer of soft carious tissue adjacent to the pulp [4]. Clinically, teeth treated with selective caries removal have excellent chances of retaining pulp vitality and increased long-term survival [5–7]. Also, this procedure can be done in a single visit unlike stepwise excavation, which requires multiple sessions and the need to re-enter the lesion [8].

The use of protective liners has been an important step in the restoration of carious lesions in close proximity to the pulp. Calcium hydroxide is the most frequently utilized protective liner in deep cavities. Its alkaline pH, capacity to stimulate the growth of reparative dentin, antimicrobial qualities, and biocompatibility signal remineralization at the interface between the pulp and dentine [9, 10]. However, it has a poor seal and is extremely soluble, vulnerable to dissolution over time [10]. As a result, several alternative materials have started to be employed, like glass ionomer cement (GIC), resin-modified glass ionomer cement (RMGIC), and calcium silicate cements such as Biodentine, mineral trioxide aggregate (MTA), and Theracal. GIC bonds chemically to the tooth and has fluoride-releasing properties. RMGIC has improved mechanical properties, less moisture sensitivity, and decreased microleakage [11]. However, its biocompatibility is questioned due to the release of hydroxyethyl methacrylate (HEMA), which is cytotoxic to pulpal cells [12, 13]. Newer calcium silicate materials such as MTA and Biodentine have bioactive properties by release of growth factors, which promote hard tissue formation [14]. Biodentine has good handling properties and promotes reparative dentin formation like MTA. It also acts as a dentine substitute and does not induce tooth discoloration compared with MTA [15]. However, it has poor bonding to the overlying resin-based restoration due to its water-based chemistry [15]. To overcome this drawback, the light-cured resin-modified tricalcium silicate Theracal LC was introduced. It has been shown to release more calcium ions than dycal, which promotes the formation of mineralized hard tissues [16]. Theracal has lower solubility and displays higher bond strength to composite [17]. One of the drawbacks is its cytotoxicity to odontoblasts, which is attributed to the presence of monomers such as bisphenol A-glycidyl methacrylate (BisGMA), HEMA, triethylene glycol dimethacrylate (TEGDMA), and urethane-dimethacrylate (UDMA) [18].

All of the materials discussed above have some limitations as protective liners after selective caries removal, and their superiority over one another is yet to be proven. It is quite confusing as there is enormous literature on this topic with no definite conclusions. A critical point of debate in clinical decision-making following selective caries removal is the necessity and efficacy of placing a liner beneath the final restoration. Proponents of liner use argue that materials such as calcium hydroxide, MTA, or GICs can promote pulpal healing, stimulate tertiary dentin formation, and enhance remineralization of the residual carious dentin. In contrast, a growing body of evidence suggests that the long-term success of restorations placed after selective caries removal is more dependent on achieving a well-sealed restoration rather than the use of an intermediary liner. This divergence in clinical practice highlights the need to critically evaluate the existing evidence regarding the efficacy of various protective liners. Thus, this systematic review aims to critically evaluate the clinical and radiographic success of various protective liners in preserving pulp vitality after selective caries removal in permanent posterior teeth.

## Materials and methods

### Protocol and registration

The study protocol was registered in the International Prospective Register of Systematic Reviews (PROSPERO) database with the following registration no. CRD42023416352 and was conducted according to Preferred Reporting Items for Systematic Review and Meta-analysis (PRISMA) [19].

### Eligibility criteria and search strategy

The following PICOS strategy was constructed:

Population (P) included patients with deep carious lesions in permanent posterior teeth, which required indirect pulp capping; Intervention (I) was placement of GIC or RMGIC or MTA or Biodentine or Theracal as a protective liner after selective caries removal; Comparison (C) was placement of calcium hydroxide as a protective liner after selective caries removal; Outcome (O) was pulp vitality determined clinically and radiographically after a minimum of 1-year follow-up, and study design (S) included was only randomized controlled trials. Studies published in the English language from inception to July 2024 were included.

Case reports, animal studies, in vitro studies, and review papers were also excluded.

### Search strategy

Four databases such as PubMed, Cochrane Central Register of Controlled Trials, ScienceDirect, Web of Science, and Google Scholar were electronically searched by two authors (VP and AJ). To find potential new studies, manual journal-specific area searches, searches of relevant reviews, and searches of cross references were added to the electronic search. Keywords were used in the search for articles. The MeSH terms used in the search are given in Appendix 1.

### Study selection and data extraction

Two reviewers (VP and AJ) independently examined titles and abstracts to select the studies manually. After that, the full texts of all the studies that qualified were obtained and examined by the two reviewers. A Microsoft Excel spreadsheet was created by extracting the pertinent data from the included studies.

Data extraction consisted of study characteristics (year, author, country, design of study), sample characteristics (sample size, age of participants, tooth), methodological details (type of protective liners, intervention, comparison, final restoration, follow up, funding), outcome (success, failures, conclusion), and measurement methods (pulp vitality clinically and radiographically). Details have been given in Tables 1–5.

**Table 1:** RMGIC versus Calcium hydroxide.

Sr. No	Author, Year, & Country	Study design	Tooth	Sample size	Age group	Intervention	Comparison	Outcome	Success intervention	Success comparison	Failure intervention	Failure comparison	Dropout intervention	Dropout Comparison	Funding	Final restoration	Conclusions
									12 months	12 months	12 months	12 months					
1	Singh S et al. 2019 India [24]	RCT parallel double blind	Permanent molars	132	14–54 years	RMGIC (GC II Fuji Lining LC) *n* = 66	CH liner (Dycal; Dentsply/Caulk *n* = 66	Pulp vitality	55/57	61/63	2	2	9	3	None	Composite	Success of the treatment is independent of the lining material used over the demineralized dentin.
2	Kaul S et al. 2021 India [25]	RCT	Permanent molars	48	12–18 years	RMGIC (GC Fuji II LC) *n* = 24	CH liner (Dycal; Dentsply/Caulk *n* = 24	Pulp vitality	21/24	22/24	3	2	0	0	None	Composite	Calcium hydroxide exhibited higher success rate as compared with RMGIC but with no statistically significant difference.

CH: calcium hydroxide; RMGIC: resin-modified glass ionomer cement; RCT: randomized controlled trial.

**Table 2:** Biodentine versus calcium hydroxide.

Sr. No	Author, Year, & Country	Study design	Tooth	Sample size	Age group	Intervention	Comparison	Outcome	Success intervention	Success comparison	Failure intervention	Failure comparison	Dropout intervention	Dropout Comparison	Funding	Final Restoration	Conclusion
									12 months	12 months	12 months	12 months					
1	Rahman B et al. 2021 [26]	RCT	Permanent posteriors	40	7–15 years	Biodentine (septodont) *n* = 20	CH liner (Dycal; Dentsply/Caulk *n* = 20	Pulp vitality	18/19	17/19	1	2	1	1	None	Composite	The success rate of calcium hydroxide is lower in comparison to Biodentine but with no statistically significant difference.
2	Kaul S et al.2021 [25]	RCT	Permanent molars	48	12–18 years	Biodentine (septodont) *n* = 24	CH liner (Dycal; Dentsply/Caulk *n* = 24	Pulp vitality	23/24	22/24	1	2	0	0	None	Composite	Biodentine exhibited the highest success rate as compared with calcium hydroxide but with no statistically significant difference.

CH: calcium hydroxide; RCT: randomized controlled trial.

**Table 3:** Theracal versus Calcium hydroxide.

Sr. No	Author, Year, & Country	Study design	Tooth	Sample size	Age group	Intervention	Comparison	Outcome	Success intervention	Success comparison	Failure intervention	Failure comparison	Dropout intervention	Dropout Comparison	Funding	Final restoration	Conclusion
									12 months	12 months	12 months	12 months					
1	Rahman B et al. 2021 India [26]	RCT	Permanent posteriors	40	7–15 years	Theracal LC (BiscoInc) *n* = 20	CH liner (Dycal; Dentsply/Caulk *n* = 20	Pulp vitality	19/19	17/19	0	2	1	1	None	Composite	The success rate of calcium hydroxide is lower in comparison to Theracal with no statistically significant difference.
2	Betamar N et al. 2020 Benghazi, Libya [27]	RCT	Permanent posteriors	132	18–55 years	Theracal LC (BiscoInc) *n* = 65	CH liner (Dycal; Dentsply/Caulk *n* = 67	Pulp vitality	64/65	65/67	1	2	0	0	Not mentioned	Composite	Success of IPC treatment is independent of the pulp capping material.
3	Semprum-Clavier A 2024 USA [28]	RCT	Permanent posteriors	60	Not mentioned	Theracal LC (BiscoInc) *n* = 30	CH liner (Dycal; Dentsply/Caulk *n* = 30	Pulp vitality	29/30	30/30	1	0	0	0	Not mentioned	Composite	There are no differences in the clinical outcomes between the two materials.

**Table 4:** Biodentine versus GIC.

Sr. No	Author and Year	Study design	Tooth	Sample size	Age group	Intervention	Comparison	Outcome	Success intervention	Success comparison	Failure intervention	Failure comparison	Dropout intervention	Dropout Comparison	Funding	Final Restoration	Conclusion
									12 months	12 months	12 months	12 months					
1	Hashem D et al. 2015 UK [29]	RCT	Permanent posteriors	72	18 to 76 years	Biodentine (Septodont) *n* = 36	GIC (Fuji IX) *n* = 36	Pulp vitality	27/33	27/33	6	6	3	3	Yes	Composite	Although no statistically significant difference was detected in the clinical efficacy of Biodentine/Fuji IX in patients with reversible pulpitis, CBCT showed a significant difference in that most healed CBCT lesions had received Biodentine while most that did not heal received Fuji IX.

CH: calcium hydroxide; GIC: glass ionomer cement; RCT: randomized controlled trial.

**Table 5:** MTA versus Calcium hydroxide.

Sr. No	Author and Year	Study design	Tooth	Sample size	Age group	Intervention	Comparison	Outcome	Success intervention	Success comparison	Failure intervention	Failure comparison	Dropout intervention	Dropout Comparison	Funding	Final Restoration	Conclusion
									12 months	12 months	12 months	12 months					
1	Koc Vural U et al. 2017 Turkey [30]	RCT	Permanent posteriors	100	Mean age 21 years	MTA (Dentsply Tulsa Dental) *n* = 51	CH (Dycal, Dentsply) *n* = 49	Pulp vitality	50/51	45/49	1	4	0	0	Yes	Composite	Both materials were found to be clinically acceptable at 12 months post-treatment with no significant differences.
2	Sultana R et al. 2016 Bangladesh [31]	RCT	Permanent teeth	50	16–30 years	MTA (Proroot Dentsply Tulsa Dental) *n* = 25	CH powder (Deepti Dental Product, India) + Saline *n* = 25	Pulp vitality	24/25	19/25	1	6	0	0	Not mentioned	Composite	Clinical outcomes and conduction of reparative dentin by MTA are more effective than that of calcium hydroxide with a statistically significant difference.

CH: calcium hydroxide; MTA: mineral trioxide aggregate; RCT: randomized controlled trial.

### Risk of bias in individual trials

The quality of the included trials was evaluated by two reviewers (VP and AJ) independently using the Cochrane risk of bias tool RoB 2 in five domains [20]: bias resulting from the randomization process, bias resulting from deviations from intended interventions, bias resulting from missing outcome data, bias in measuring the outcome, and bias in selecting the reported result. The algorithms of the RoB 2 tool map responses to signaling questions on a potential risk of bias judgment for each outcome evaluated [20]. Every study was categorized according to three risk levels: low, some concerns, and high. Studies were classified into low risk if all domains were deemed to be low risk; some concerns when one of the domains was determined to raise some concern; high risk when a trial had at least one domain with a high risk of bias or when one or more domains were determined to raise concerns.

### Certainty of evidence assessment

The GRADE (Grading of Recommendations, Assessment, Development, and Evaluations) approach was used for assessing the quality of evidence in our systematic review. The quality of evidence was classified into high, moderate, low, and very low based on serious factors like limitations in study design, risk of bias, inconsistency of results, indirectness of evidence, imprecision, and publication bias [21].

### Data synthesis

The RevMan (Review Manager version 5.4.1) software was used to conduct the meta-analysis. The findings of each treatment group were pooled together using the risk ratio (RR) within the 95% confidence interval (CI) for dichotomous outcomes, such as pulp vitality (success or failure). The effect was judged significant if the *P* value was less than 0.05. The Cochran’s Q statistic and the *I*^2^ statistic were used to assess heterogeneity among the included studies. Significant heterogeneity was defined as *I*^2^ values greater than 50%. Fixed-effects model was employed when no substantial heterogeneity was observed, while random-effects model was used when considerable heterogeneity was detected [22]. Only studies with similar outcomes were subjected to meta-analysis. Because the included studies reported pulp vitality with different lengths of follow-ups, the meta-analysis focused on the success rates at 1-year follow-up.

### Publication bias

Publication bias could not be assessed by using a funnel plot due to a low number of studies included in our meta-analysis. The power of tests is extremely low when there are few studies in order to differentiate chance from actual asymmetry [23].

## Results

### Study selection

**Figure 1:**
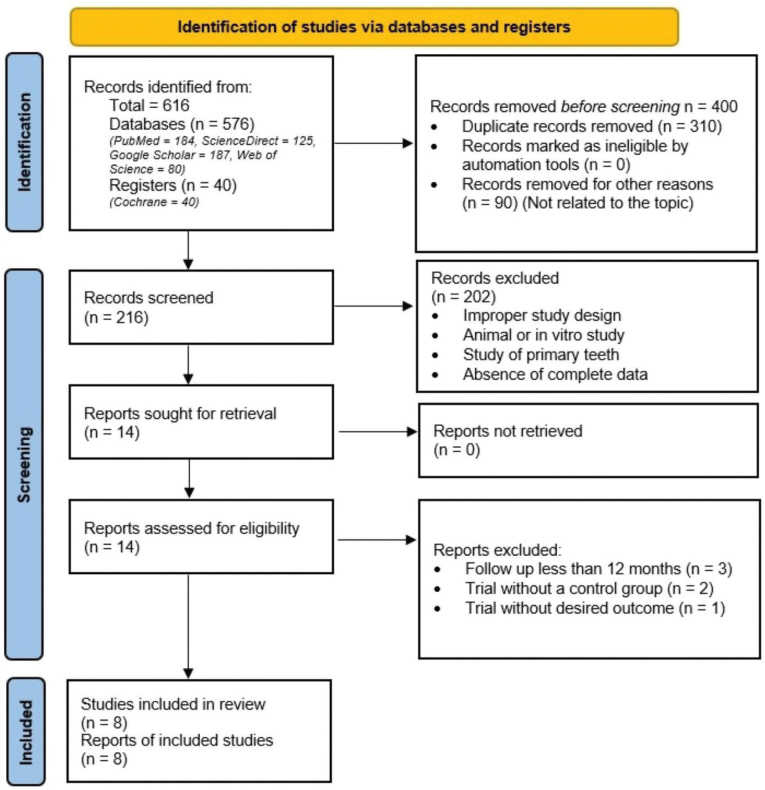
PRISMA 2020 flow diagram for new systematic reviews, which included searches of databases and registers only.

A search of the five databases generated 616 results. Date of last search was 01/08/2024. After removing 310 duplicates and 90 unrelated articles, a total of 216 articles were included in title and abstract screening. 202 articles that addressed stepwise or complete caries excavation, direct pulp capping, studies on primary teeth, studies with improper study design, animal or in vitro studies, studies with absence of complete data, and unclear methodology were excluded. 14 full-text articles remained to be examined for eligibility. Six articles were excluded after full-text evaluation as they lacked a comparison group; analyzed different outcomes such as microbiologic, ultrastructural, or histologic; and had a follow-up of less than 1 year. Finally, a total of eight articles were included [24–31]. The Cohen’s kappa value for the selection of studies was 1, which indicated perfect agreement. Our study selection process is shown in Figure 1.

### Study characteristics

The main characteristics and outcomes of the included studies are summarized in Tables 1–5. A total of 678 teeth were subjected to selective caries removal and received application of various protective liners. All of the eight studies were randomized controlled trials from different countries. All included trials had primary deep occlusal or occlusal-proximal caries involving at least two-thirds of dentine detected radiographically. Vital teeth with reversibly inflamed pulp, a positive response to cold test without any periapical, or periodontal conditions were included. The included trials assessed pulp vitality after a period of 1 year. One trial treated the cavity base with 2% chlorhexidine gluconate for 60 s followed by RMGIC [25]. Another trial used CBCT along with periapical radiographs at baseline and 12 months follow-up to detect periapical changes [29]. Radiological outcomes denoted by reparative dentin formation were evaluated in a trial with periapical radiographs [31]. Pulp vitality was assessed as success or failure by a combination of clinical and radiographic examinations. A positive response to cold test; negative response to percussion; and absence of pain, abscess, or sinus tract were considered clinical success. The lack of periapical disease or alterations like loss of lamina dura, periodontal ligament space widening, root canal obliteration, and resorption were used to determine radiographic success. In all studies, clinical and radiographic assessments were conducted prior to, immediately following treatment, and at follow-up visits.

### Risk of bias

**Figure 2:**
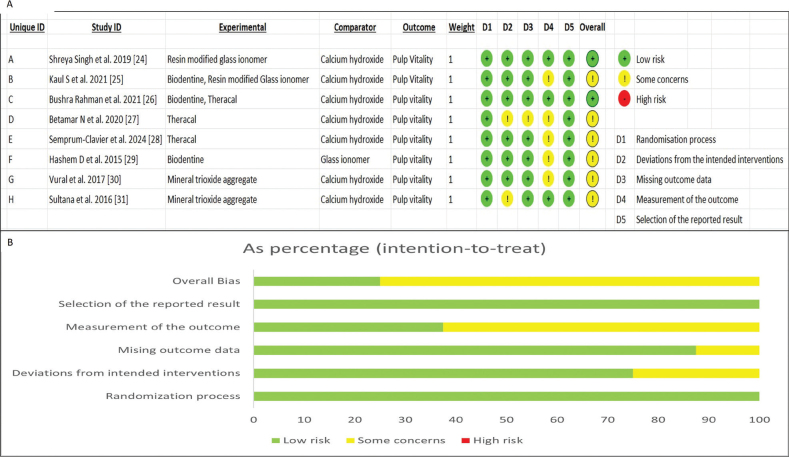
Risk of bias. (A) Risk of bias ROB 2 tool. (B) Risk of bias ROB 2 tool summary of each domain.

The risk of bias assessment is presented in Figure 2A, B. All eight trials gave a clear idea regarding the process of randomization. Allocation concealment was clearly addressed in all trials [24–31]. Regarding the blinding of participants and operator, it was impossible to conceal the treatment method from the operator. There were deviations from the intended interventions in two trials [27, 31]. Bias aroused due to missing outcome data in one of the trials [27]. There was bias in the measurement of outcome in five trials. Blinding to outcome assessment was not clear in five trials [25, 27–30]. No trials had deviations in the selection of the reported result. Overall, two included studies were classified as low risk, [24, 26] and six studies were having some concerns [25, 27–31].

### Certainty of evidence assessment

**Figure 3:**
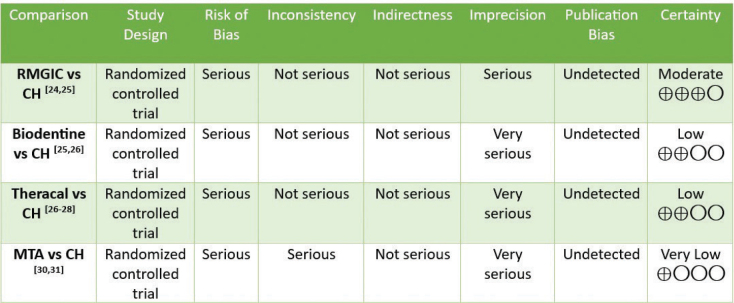
Rating the certainty of evidence by GRADE (Grading of Recommendations Assessment, Development, and Evaluation) tool.

The quality of evidence was graded as moderate for one comparison between RMGIC and calcium hydroxide [24, 25]; low for two comparisons between Biodentine and calcium hydroxide [25, 26] and Theracal and calcium hydroxide; [26–28] and very low for comparison between MTA and calcium hydroxide [30, 31]. The authors suggest that the true effect size may differ considerably from the estimated effect. Only randomized trials, which provide high-quality data, were used as the study design. Due to effective blinding, allocation concealment, and comprehensive reporting of results, the risk of bias was not serious in two studies. Conversely, six studies had deviations from intended outcomes, missing outcome data, and bias in measurement of outcome. Thus, the risk of bias was serious in six studies. Statistical heterogeneity across some studies was 0.00%, but there were methodological differences contributing to clinical heterogeneity. One of the comparisons between MTA and calcium hydroxide demonstrated heterogeneity of 54%. Thus, inconsistency was rated as serious for one comparison studies [30, 31]. As the results of studies were based on direct comparisons, there was no indirectness. Population, intervention, and outcome measures did not significantly vary between trials. As there were serious discrepancies in sample sizes, the degree of imprecision was rated as serious for one comparison [24, 25] and very serious for three comparisons [25–31]. Publication bias could not be determined as there were few studies. Details have been shown in Figure 3.

### Meta-analysis

**Figure 4:**
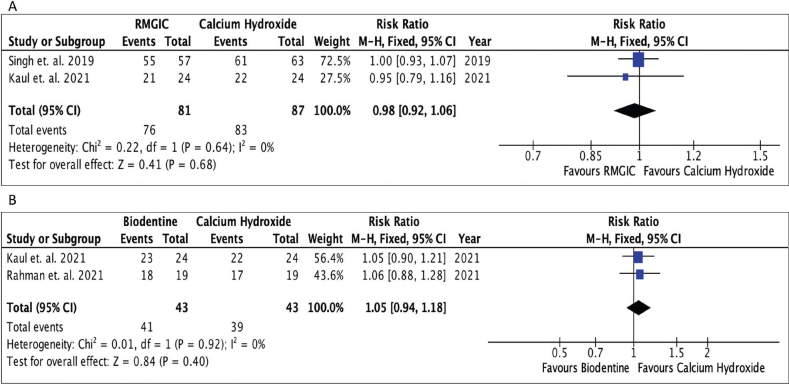
Forest plots. (A) Forest plot comparing RMGIC and calcium hydroxide; (B) Forest plot comparing Biodentine and calcium hydroxide.

**Figure 5:**
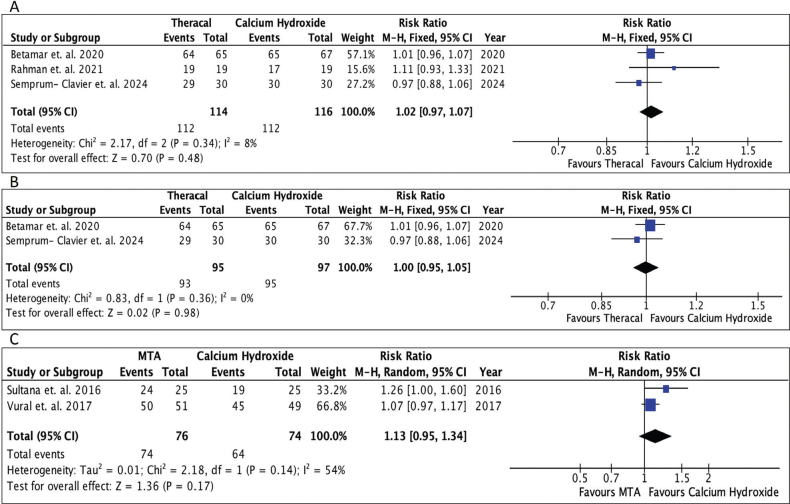
Forest plots. (A) Forest plot comparing Theracal and calcium hydroxide. (B) Sensitivity analysis (Theracal and calcium hydroxide). (C) Forest plot comparing MTA and calcium hydroxide.

The final meta-analysis included four comparison groups: 1. RMGIC and calcium hydroxide, 2. Biodentine and calcium hydroxide, 3. Theracal and calcium hydroxide, and 4. MTA and calcium hydroxide. As there was a single study comparing Biodentine and GIC, it was not subjected to meta-analysis. Standard pair-wise meta-analysis of direct comparisons between each group was carried out. The RR for RMGIC and calcium hydroxide was 0.98 (95% CL = 0.92 to 1.06; *P* = 0.68), indicating that there was no significant difference between RMGIC and calcium hydroxide in preserving pulp vitality at 1-year follow-up (Figure 4A). The RR for Biodentine and calcium hydroxide was 1.05 (95% CL = 0.94 to 1.18; *P* = 0.40), indicating that there was no significant difference in clinical outcomes between Biodentine and calcium hydroxide at 1-year follow-up (Figure 4B). The RR for Theracal and calcium hydroxide was 1.02 (95% CL = 0.97 to 1.07; *P* = 0.48), indicating that there was no significant difference in clinical outcomes between Theracal and calcium hydroxide at 1-year follow-up (Figure 5A). Sensitivity analysis was also done by eliminating the study by Rahman et al. due to its wider CI and serious risk of bias (Figure 5B). The results obtained were nonsignificant. The RR for MTA and calcium hydroxide was 1.13 (95% CL = 0.95 to 1.34; *P* = 0.17), indicating that there was no significant difference in clinical outcomes between MTA and calcium hydroxide at 1-year follow-up (Figure 5C). There was moderate heterogeneity of 54% for the comparison between MTA and calcium hydroxide. A random-effects model was employed in the meta-analysis to account for potential variability in true effect sizes among studies arising from differences in study populations, interventions, settings, and methodologies.

## Discussion

This systematic review and meta-analysis included trials that compared calcium hydroxide, GIC, resin-modified glass ionomer, MTA, Biodentine, and Theracal in patients with deep caries after selective caries removal. This review revealed no statistically significant differences between RMGIC, calcium hydroxide, Biodentine, Theracal, and MTA in maintaining pulp vitality. It can be concluded that there was no significant difference in clinical and radiographic outcomes between calcium hydroxide, RMGIC, Biodentine, Theracal, and MTA when used as a protective liner. Thus, the type of protective liner used does not influence treatment success after selective caries removal. Nevertheless, these findings should be interpreted with caution due to the limited number of included studies and the low to moderate certainty of the supporting evidence.

Deep carious lesions are more challenging to treat as there is a higher chance of pulp exposure in these cases, which makes the treatment outcome less predictable. Protective liners have been suggested after selective caries removal as they are able to remineralize the affected dentin and promote reparative dentin formation. But the role of these agents has been questioned by some studies [32, 33]. Studies have shown that the liner application does not influence success rates [32, 33]. Direct composite resin restorations without liner application in patients with deep caries have shown no difference in clinical outcomes when compared with application of liners [24]. This suggests that there is poor evidence about the role of liners in deep caries. Also, the type of protective liner seems to have no significant differences in clinical outcomes at 1-year follow-up, in spite of their bioactive, remineralizing, and antibacterial properties [24]. Evidence suggests that after removal of maximum amount of caries (the major source of infection) peripherally and centrally, leaving a layer of soft caries over the pulp (to prevent pulp exposure) and obtaining a good interfacial seal by a satisfactory restoration are sufficient for isolating the microorganisms involved in the carious process and their subsequent entombment, depriving them of their nutrition supply, thus halting the caries progression, promoting the host response for self-repair (reactionary or reparative dentin), and preserving pulp vitality [24, 32].

Calcium hydroxide has for long been used as a liner due to its proven antibacterial and hard tissue formation properties [9]. However, dentin bridges formed by calcium hydroxide often contain tunnel defects and cell inclusions, resulting in inadequate sealing and microleakage [9]. Moreover, it is water soluble and has been reported to degrade within 6 months, leaving voids beneath the restoration that act as conduits for pathogenic bacteria and ultimately recurrent pulpal inflammation and necrosis [34]. Also, these voids may cause dentinal fluid flow, resulting in dentin hypersensitivity. Long-term clinical investigations have demonstrated that calcium hydroxide fails more frequently over time due to its incapability to closely adapt to dentin, gradual degradation over time, inability to consistently support odontoblast development, and cytotoxicity [35]. Another clinical parameter that requires consideration is that calcium hydroxide cannot be acid etched; thus it requires a GIC base followed by composite [36]. Thus, to surpass the limitations of calcium hydroxide, other materials are used like GIC and RMGIC. Nevertheless, they cannot be used when the RDT is ≤ 0.5 mm in order to prevent pulp injury [12].

Bioactive materials such as MTA and Biodentine were introduced, which are known to promote hard tissue formation by release of growth factors transforming growth factor beta 1 (TGF-b1) [37]. A study revealed higher success with MTA (96%) compared with calcium hydroxide (76%). Also, it assessed reparative dentin formation at 12 months. 76% of teeth treated with calcium hydroxide, and 96% of MTA-treated teeth showed reparative dentin formation [31]. A study reported greater clinical success and efficacy of MTA and Biodentine compared with calcium hydroxide [38]. Another study showed that Biodentine exhibited a similar effect on dentin bridge formation as MTA [39]. A drawback of MTA is its slower setting time, which requires a two-sitting approach [14]. MTA (Angelus) has a shorter setting time of 15 min, which provides an advantage for the placement of definitive restoration immediately. A study reported reduced compressive strength and surface microhardness after acid etching of MTA. They suggested to delay the final restorative procedure for at least 96 h [40]. In a recent research, MTA demonstrated the highest bond strength with two-step SE group at 15 min rather than 72 h [41]. They attributed it to the porosity of MTA, which would have facilitated better penetration of adhesives within this time interval [41]. Thus, a single sitting procedure with placement of final restoration could be accomplished with MTA and has been supported by studies [42]. In the above-mentioned study, the highest bond strength for Biodentine was observed with total-etch and two-step SE adhesives bonded at 72-h interval [41]. This could be as a result of the porous nature of Biodentine, which requires time for the hydrated calcium silicate gel to crystallize and reach a bulk strength that allows it to withstand the polymerization stresses [41]. Biodentine has shown more effective reparative dentin formation compared with calcium hydroxide [43]. In our systematic review and meta-analysis, we found no significant differences in clinical outcomes with Biodentine and calcium hydroxide. This could be due to low number of studies included and variations in study design.

A recent systematic review and meta-analysis evaluating the outcomes of vital pulp therapy in permanent teeth concluded that, over a 24-month follow-up period, there was no statistically significant difference in the success rates of indirect pulp treatment when using GIC, calcium hydroxide, or calcium silicate-based cements [44]. This finding was consistent among teeth diagnosed with either normal pulp or reversible pulpitis, suggesting that the choice of pulp capping material may not critically impact the long-term clinical success of indirect pulp treatment in such cases [44]. A systematic review and meta-analysis concluded that partial caries removal, with or without a liner, yields comparable outcomes in pulp vitality after 1 year [45]. The role of liners in such therapy remains controversial. By eliminating necrotic dentine and isolating residual bacteria from the oral environment, a proper coronal seal can impede bacterial metabolism and support the dentine-pulp complex’s natural defense and healing responses [45]. Another study included concluded that Theracal has higher success rate than calcium hydroxide [26]. Theracal releases more Ca ions than Dycal and has apatite forming ability [16]. The advantage of Theracal over all other agents is that it can be light cured, thus allowing immediate placement of final restoration without any contamination [46]

The studies included in our systematic review evaluated pulp vitality based on no symptoms, positive response to cold test and by radiographs. According to a study, cold test was the most accurate method for sensitivity testing [47]. Cold tests are not completely reliable, and a definitive diagnosis can only be achieved after a histologic examination [48]. Thus, the information collected should be interpreted carefully as teeth with pulp necrosis could be asymptomatic. Also, teeth that are responsive may have portions of pulp, which are irreversibly damaged [48]. Hence, it is likely that incorrect diagnosis would have led to bias. It would be better to use a combination of tests for diagnosis and evaluation of outcome like electric pulp test along with cold test until methods with greater specificity and sensitivity are adopted.

Another suggestion that can be considered while designing future trials is to define the volume of residual caries left. None of the studies quantified the residual volume or thickness of caries. The amount of soft caries remaining over the pulp could affect the pulp vitality and restoration survival. Additional variables like hardness of dentin (soft, firm or leathery), along with removal methods, (micromotor, spoon excavator, turbine) should be standardized [6].

According to a systematic review, selective caries removal to soft dentin may increase restoration failure [49]. The demineralized dentin could reduce adequate bonding to the overlying restoration [50]. Thus, it can be suggested to recall patients at shorter intervals to evaluate restoration quality and control caries.

The strengths of this systematic review are as follows: an a priori protocol was developed and registered in PROSPERO, a thorough literature search was conducted, and there was minimal statistical heterogeneity across studies. The moderate number of studies included in qualitative and quantitative analysis is one of the potential limitations of this review. Sample sizes in individual trials were small (mean per group often < 50). Clinical heterogeneity, which included residual dentin thickness, restoration type, and diagnostic tests, was acknowledged but not quantitatively analyzed in any of the trials. Meta-regression or subgroup analysis was not feasible due to insufficient data or inconsistent reporting across studies. The risk of bias was serious in the majority of studies along with very low, low, and moderate certainty of evidence. This weakens pooled conclusions. Some trials included adjunctive agents (e.g. 2% chlorhexidine), which may have confounded the results [25]. In addition, this review included papers in the English language only, which may have led to reporting bias. Exclusion of nonrandomized trials may have limited the evidence pool for rare materials like Theracal. Also, publication bias was not assessed due to a low number of studies. The level of research and number of the studies included in this systematic review suggest that more randomized clinical trials with larger sample sizes, standardized outcome measurement methods (residual dentin thickness measurement, quantification of residual caries, histological confirmation), and longer follow-up periods must be carried out to evaluate the effect of protective liners on pulp vitality, restoration quality, and longevity following partial caries removal in permanent teeth.

## Conclusions

Within the limitations of this study, it can be concluded that

There is no difference in efficacy of RMGIC and calcium hydroxide, Biodentine and calcium hydroxide, Theracal and calcium hydroxide, and MTA and calcium hydroxide in preserving pulp vitality after selective caries removal in permanent teeth.

The preservation of the pulp’s vitality and the success of treatment are not significantly affected by the types of liner material evaluated. However, these findings should be interpreted with caution due to the low certainty of the evidence.

## APPENDICES

### Appendix

**1**: MESH terms

 APPENDIX 1

 Date of last search: 01/08/2024

 The following MeSH terms, search terms, and their combinations were used in the search:

 PubMed (184)

 (“Dental Caries”) AND (‘‘Dental Cavity Lining OR Liners’’) AND (‘‘Dental Pulp) AND (‘‘Treatment Outcome”) AND (‘‘Permanent’’) AND (“Adult”) AND (“Dental Restoration”) AND (“Calcium Hydroxide”) AND (“Glass Ionomer Cements”) AND (“Calcium silicate”) AND (“mineral trioxide aggregate”) AND (“tricalcium silicate”) AND (“Dental Pulp Test”) AND (“Pulp Outcome OR Pulp Vitality’’) AND (“Partial Caries removal”) AND (“Deep caries OR Deep caries Management”) AND (“Incomplete caries removal”) AND (“Selective caries removal”) AND (“Peripheral caries removal’’) AND (“Indirect pulp capping”)

 ScienceDirect (125)

 (“Dental Caries”) AND (“Incomplete caries removal”) AND (“Indirect pulp capping”) OR (“Dental Pulp Capping”) AND (“Glass Ionomer Cements”) AND (“Calcium Hydroxide”) AND (“Calcium silicate”) OR (“mineral trioxide aggregate”) OR (“tricalcium silicate”) AND (“Pulp Outcome OR Pulp Vitality’’) AND (“Dental Cements”)

 Web of Science (80)

 (“Calcium Hydroxide”) AND (“Glass Ionomer Cements”) AND (“Calcium silicate”) AND (“mineral trioxide aggregate”) AND (“tricalcium silicate”) AND (“Dental Pulp Test”) AND (“Pulp Outcome OR Pulp Vitality’’) AND (“Partial Caries removal”) AND (“Deep caries OR Deep caries Management”) AND (“Incomplete caries removal”)

 Google Scholar (187)

 (“Silicates”) OR (“Calcium Compounds”) AND (“Dental Pulp Capping”) AND (“Treatment Outcome”) AND (“Selective caries removal”) AND (“Peripheral caries removal’’) AND (“Indirect pulp capping”) AND (“Calcium Hydroxide”) AND (“Glass Ionomer Cements”)

 Cochrane (40)

 (“Glass Ionomer Cements”) AND (“Calcium Hydroxide”) AND (“Calcium silicate”) OR (“mineral trioxide aggregate”) OR (“tricalcium silicate”) AND (“Pulp Outcome OR Pulp Vitality’’) AND (“Dental Cements”)
